# Complete remission of primary plasma cell leukemia with bortezomib, doxorubicin, and dexamethasone: a case report

**DOI:** 10.1186/1757-1626-2-121

**Published:** 2009-02-04

**Authors:** Steven M Chan, Tracy George, Athena M Cherry, Bruno C Medeiros

**Affiliations:** 1 Division of Hematology, Department of Medicine, Stanford University School of Medicine, Stanford, CA, USA; 2 Department of Pathology, Stanford University School of Medicine, Stanford, CA, USA

## Abstract

**Background:**

Plasma cell leukemia (PCL) is a rare lymphoproliferative disorder considered to be a variant of multiple myeloma. It is an aggressive disease with a poor clinical response to standard chemotherapeutic agents.

**Case presentation:**

A novel regimen consisting of bortezomib, doxorubicin, and dexamethasone is currently under active evaluation for the treatment of multiple myeloma. We employed this combination as front-line chemoinduction therapy for a case of primary PCL.

**Conclusion:**

Complete remission was achieved with rapid normalization of hematologic parameters. The combination of bortezomib, doxorubicin and dexamethasone demonstrates promise in the treatment of PCL.

## Background

Plasma cell leukemia (PCL) is a rare but aggressive form of lymphoproliferative disorder characterized by malignant plasma cells in the bone marrow and peripheral blood. This entity is considered to be a variant of multiple myeloma (MM) that can present *de novo *in patients with no preceding MM or in patients with established MM after leukemic transformation [1]. Although PCL and MM are closely related disease entities, the prognosis of PCL patients treated with standard chemotherapy has consistently been shown to be inferior to that of MM patients [1,2]. Due to the rarity of PCL, there has been a paucity of studies evaluating optimal treatment strategies.

Bortezomib was approved for the treatment of MM in patients who have received and failed to respond to at least one prior therapy [3]. Bortezomib represents a new class of anti-neoplastic medications that selectively inhibits the activity of the 26S proteasome complex of the cell [3]. The mechanisms underlying the anti-neoplastic effects of bortezomib include NF-κB inhibition, upregulation of apoptotic pathways, and effects on the tumor microenvironment [3]. While the therapeutic efficacy of bortezomib is well established for MM, only several case reports have been published reporting efficacy of it in the treatment of PCL either as monotherapy or in combination with other agents [4-7]. A recent retrospective survey of twelve cases of PCL treated with bortezomib showed an initial response rate of 92% [8].

Here, we detail our experience in the management of a case of primary PCL. This case is interesting in several ways. From the diagnostic and prognostic perspectives, the malignant plasma cells showed an unusual "hairy-cell" morphology, harbored a t(11;14), and strongly expressed cyclin D1. From the treatment perspective, complete remission was achieved with a novel combination regimen of bortezomib, doxorubicin, and dexamethasone as first-line induction chemotherapy. This combination is currently under active evaluation for the treatment of MM [9] but has not been extensively studied for the treatment of PCL. Furthermore, this case highlights two important toxicities related to the use of high-dose bortezomib and their impact on management.

## Case presentation

**Figure 1:**
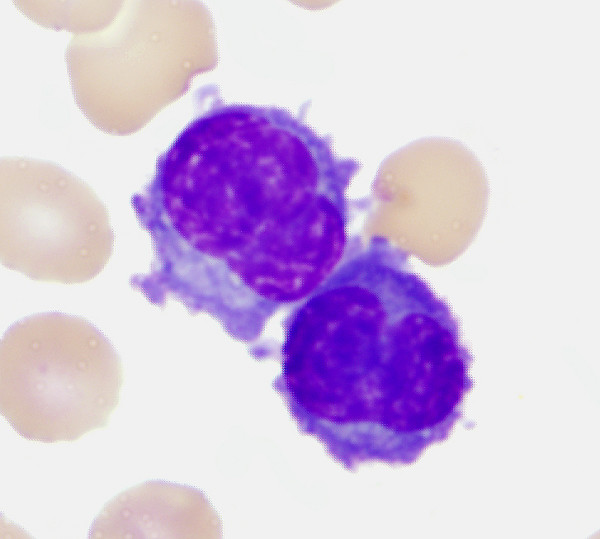
**Peripheral blood smear showing the morphology of patient's leukemic cells**.

A 54 year-old African-American man with no significant past medical history was transferred to our institution with right sided abdominal discomfort for 1 week. On admission, he noted recent onset of polyuria, anorexia, fatigue and dyspnea. Physical examination was notable for right upper quadrant abdominal tenderness and absence of lymphadenopathy or splenomegaly. An electrolyte panel revealed hypercalcemia (14 mg/dL) in the setting of an elevated creatinine level (3.2 mg/dL). He had a white blood cell count of 135,000/μL, hemoglobin concentration of 8.4 g/dL, and platelet count of 76,000/μL. Peripheral blood smear revealed an increased number of leukocytes that consisted of a population of atypical-appearing lymphoid cells of variable size and appearance with numerous cytoplasmic hairy projections and deeply basophilic cytoplasm (Figure 1). The cells did not show microscopic features characteristic of mature plasma cells. A core biopsy revealed a hypercellular bone marrow (90%) that was replaced by a population of atypical cells similar to the ones seen in peripheral blood. Flow cytometry and immunohistochemistry (IHC) studies of the cells revealed a population that was deficient in B and T cell specific markers but strongly expressed CD38, CD138 and cytoplasmic lambda light chain. Serum protein electrophoresis revealed an M-spike that was identified as free lambda chain on immunofixation. Serum lambda free light chain level was correspondingly elevated. The combined clinical and laboratory findings supported the diagnosis of plasma cell leukemia.

Additional prognostic studies included the following: beta-2 microglobulin, 6893 ng/mL (normal range: 609–2366); serum albumin, 3.8 g/dL (3.5–5.0); and lactate dehydrogenase, 191 U/L (<240). Serum immunoglobulin levels of all isotypes were low. A skeletal survey was negative. Conventional cytogenetic and interphase FISH analysis revealed a CCND1/IGH gene fusion rearrangement mediated by an unbalanced t(11;14)(q13;q32). This translocation brings the cyclin D1 oncogene in close proximity to the powerful Eμ enhancer of the IgH locus [10]. Cyclin D1 expression was correspondingly strong on IHC staining.

**Figure 2:**
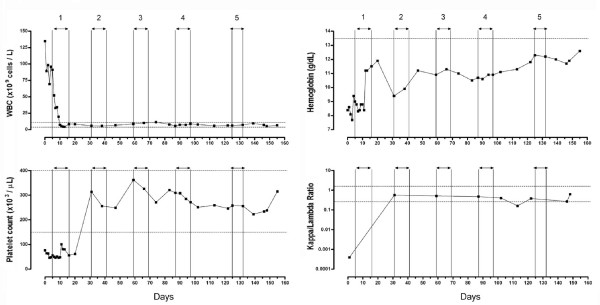
**Hematologic parameters and free kappa/lambda light chain ratio over the course of chemoinduction**. The numbers above the vertical columns represent cycles. Horizontal lines represent the lower and uppers limits of normal. In the hemoglobin graph, only the lower limit line is shown. Day "0" refers to the day of admission.

After normalization of his calcium and creatinine levels with aggressive fluid resuscitation, induction chemotherapy with bortezomib, doxorubicin, and dexamethasone was initiated. Chemotherapy was administered over the course of 5 cycles (Table 1) which resulted in a rapid suppression of the white blood cell count in association with a more gradual recovery of hemoglobin concentration and platelet count to near-normal and normal levels, respectively (Figure 2). The serum free kappa to lambda light chain ratio normalized after one cycle of chemotherapy and remained normal for the remaining cycles (Figure 2). A restaging skeletal survey revealed no interval development of bony lesions. A repeat bone marrow aspirate and biopsy showed hypocellularity (<5%) with no morphologic, cytogenetic, or immunophenotypic evidence of disease. The patient was in stringent complete remission based on the International Myeloma Working Group (IMWG) criteria [11]. At the time of publication, the patient is undergoing evaluation for either an autologous or allogeneic bone marrow transplant to consolidate treatment.

**Table 1:** Bortezomib, doxorubicin, dexamethasone chemotherapy regimen

Drug	**Cycle**^a^				
	#1	#2	#3^b^	#4	#5^c^
Bortezomib	1.3 mg/m^2^	1.3 mg/m^2^	1.0 mg/m^2^	Not given	0.7 mg/m^2^
Doxorubicin	5 mg/m^2^	5 mg/m^2^	5 mg/m^2^	4 mg/m^2^	10 mg/m^2^
Dexamethasone (IV)	40 mg	40 mg	40 mg	40 mg	40 mg (PO)

^a ^Each cycle with the exception of cycle #5 consisted of four scheduled doses on days 1, 4, 8, and 11. All three drugs were administered on each day unless otherwise stated. A cycle was started every 28 days.

^b ^Bortezomib and doxorubicin were held on cycle 3, day 11 due to progressive peripheral neuropathy.

^c ^Cycle #5 consisted of one dose of bortezomib on day 1, two doses of doxorubicin on days 1, and 8 and four doses of oral dexamethasone on days 1, 2, 8, and 9.

Chemoinduction was complicated by significant treatment related toxicities to the lung and peripheral nerves. After the first dose, the patient developed a non-productive cough without dyspnea, hypoxia or respiratory compromise. Computed tomography imaging of the chest demonstrated bilateral pleural effusions with extensive upper-lobe ground-glass opacities. Transbronchial biopsy and bronchoalveolar lavage failed to show any infectious organisms or malignancy. The pulmonary finding was therefore felt to be secondary to bortezomib which has been reported in the past [12]. The patient received a high-dose dexamethasone taper over a 4-week period which resulted in significant resolution of his cough and imaging abnormalities with no subsequent recurrence.

The patient also experienced grade 2 to 3 peripheral neuropathy which is a well documented side effect of bortezomib therapy [13]. Bilateral numbness, tingling, burning pain, and sensory loss in his toes and feet occurred soon after the first cycle. His symptoms worsened to involve motor weakness and progressed proximally to involve his lower extremities to the level of the knee after the second cycle necessitating dose reduction or deletion of bortezomib on cycles 3, 4, and 5. Trials of gabapentin, pregabalin, mexiletine, duloxetine, topiramate, amitriptyline, and methadone were attempted but all with either significant side effects or lack of symptomatic control.

## Discussion

Although PCL is a rare entity representing only 0.3% of acute leukemia cases, it is an aggressive disease with a median overall survival of less than one year with conventional chemotherapy alone [1]. Several genomic aberrations and immunohistochemical markers have been identified as prognostic indicators for MM including two markers found in our case: t(11;14) and cyclin D1 [10]. The t(11;14) which frequently results in upregulation of the oncogene cyclin D1, is correlated with a neutral or favorable prognosis in MM [10,14]. Correspondingly, cyclin D1 expression has also been associated with prolonged overall survival [10,15]. Cytogenetic abnormalities are also commonly observed in primary PCL with up to 70% of cases harboring the t(11;14)(q13;q32) [16]. A recent study by Tiedemann *et al. *reported no significant difference in overall survival between t(11;14)(q13;q32)-positive and negative PCL patients [16]. Further studies are warranted to confirm this finding and identify additional prognostic markers in PCL.

Bortezomib is effective as a single agent in the treatment of MM. Esparis-Ogando *et al*. also described the successful treatment of a patient with secondary PCL with bortezomib alone [6]. However, a highly synergistic *in vitro *anti-tumor effect between bortezomib and genotoxic chemotherapeutic agents including doxorubicin and melphalan has been shown in MM cell lines and primary MM cells [17]. This finding forms a rational basis for combination therapy in MM. Indeed, a recent phase III trial comparing bortezomib alone or in combination with pegylated liposomal doxorubicin in patients with relapsed or refractory myeloma reported an increase in median time to progression, median duration of response and 15-month survival rate [18]. Based on the above findings, we elected to use a bortezomib-based combination regimen for our patient. The combination of bortezomib, doxorubicin and dexamethasone (PAD) has previously been shown to be highly active in MM [9] but has not been extensively studied in PCL. In this report, we show that this regimen has clinical activity against t(11;14)+/cyclin D1+ PCL.

Peripheral neuropathy is a common toxicity associated with bortezomib. In a study of 256 MM patients treated with bortezomib, grade 1–2 peripheral neuropathy occurred in 22% of patients [13]. Our patient developed symptoms of neuropathy shortly after the first cycle of bortezomib. Symptoms remained even after dose reduction and discontinuation in subsequent cycles. Persistence of symptoms has been shown to occur in about 30% of patients with severe neuropathy [13]. As illustrated in this case, achieving symptomatic relief can be a challenging process requiring empiric trials of many classes of medications.

Unlike peripheral neuropathy, severe pulmonary complications occur rarely with bortezomib and have only been reported in several case reports [12,19,20]. It is of interest that many of the reported cases have occurred in patients of Japanese origin suggesting a genetic predisposition to this complication [12,19]. Our patient who is African-American developed a non-productive cough, pleural effusion and ground-glass opacities on CT imaging shortly after his first dose of bortezomib. His symptoms and imaging abnormalities responded favorably and quickly to a high-dose dexamethasone taper. The clinical course of our patient was remarkably similar to that of prior reported cases [12,19,20]. Our experience further supports the use of high-dose steroids early in the management of pulmonary complications caused by bortezomib.

This case demonstrates that the combination of bortezomib, doxorubicin, and dexamethasone is relatively well-tolerated and has clinical utility in the treatment of t(11;14)-positive plasma cell leukemias. Further evaluation of the combination in this setting is warranted.

## Consent

Written informed consent was obtained from the patient for publication of this case report and accompanying images. A copy of the written consent is available for review by the Editor-in-Chief of this journal.

## Competing interests

The authors declare that they have no competing interests.

## Authors' contributions

SMC gathered patient's data and prepared the manuscript. TG and AMC helped with the analysis and interpretation of data. BCM was involved in the management of this case and preparation of the case report.
